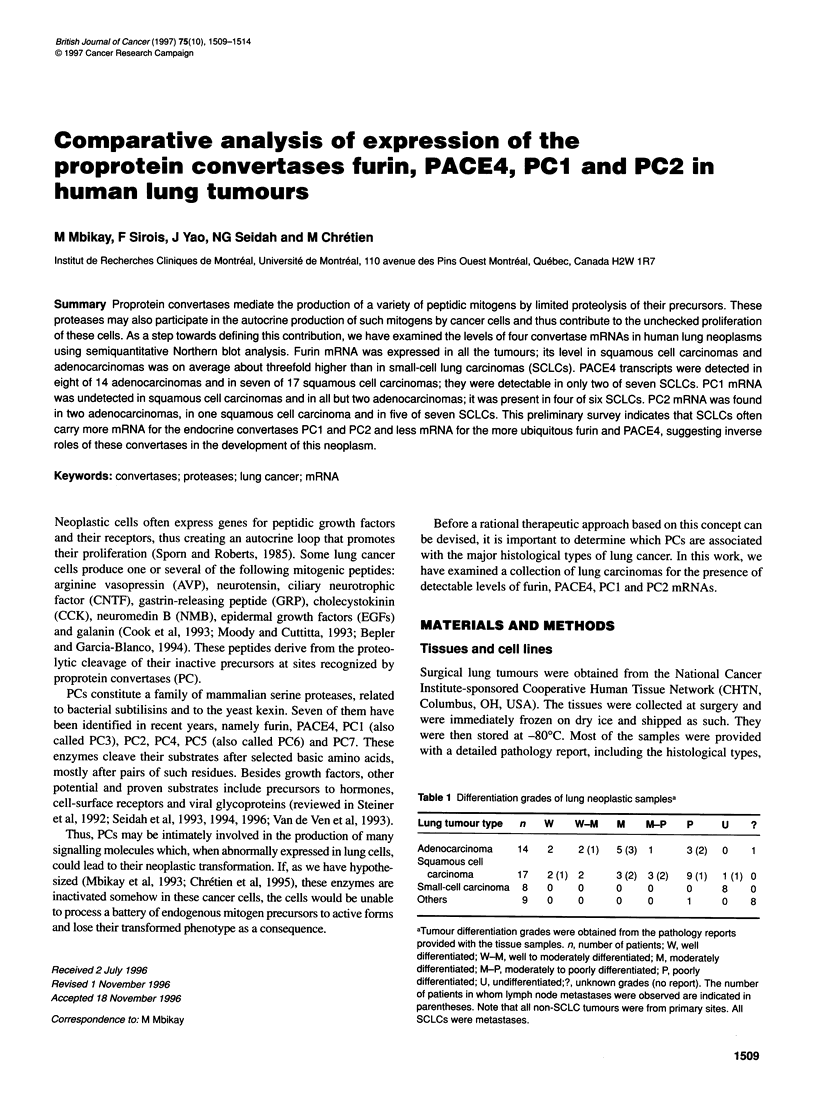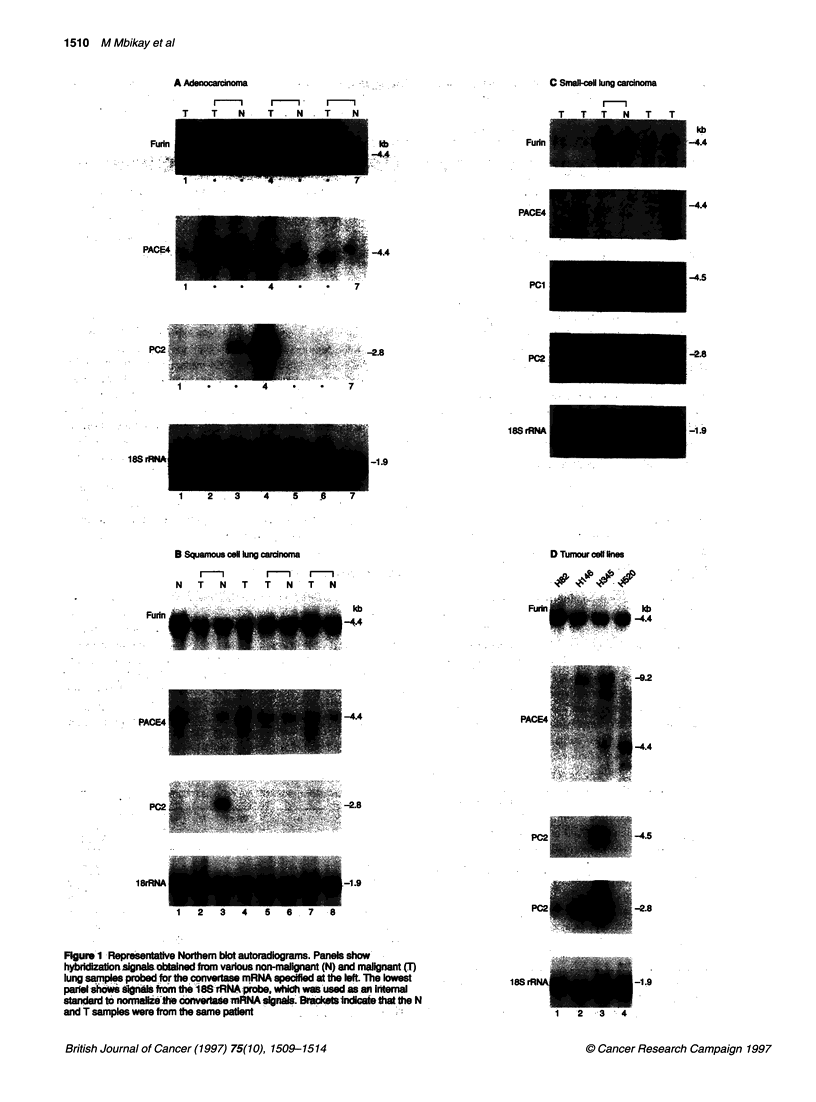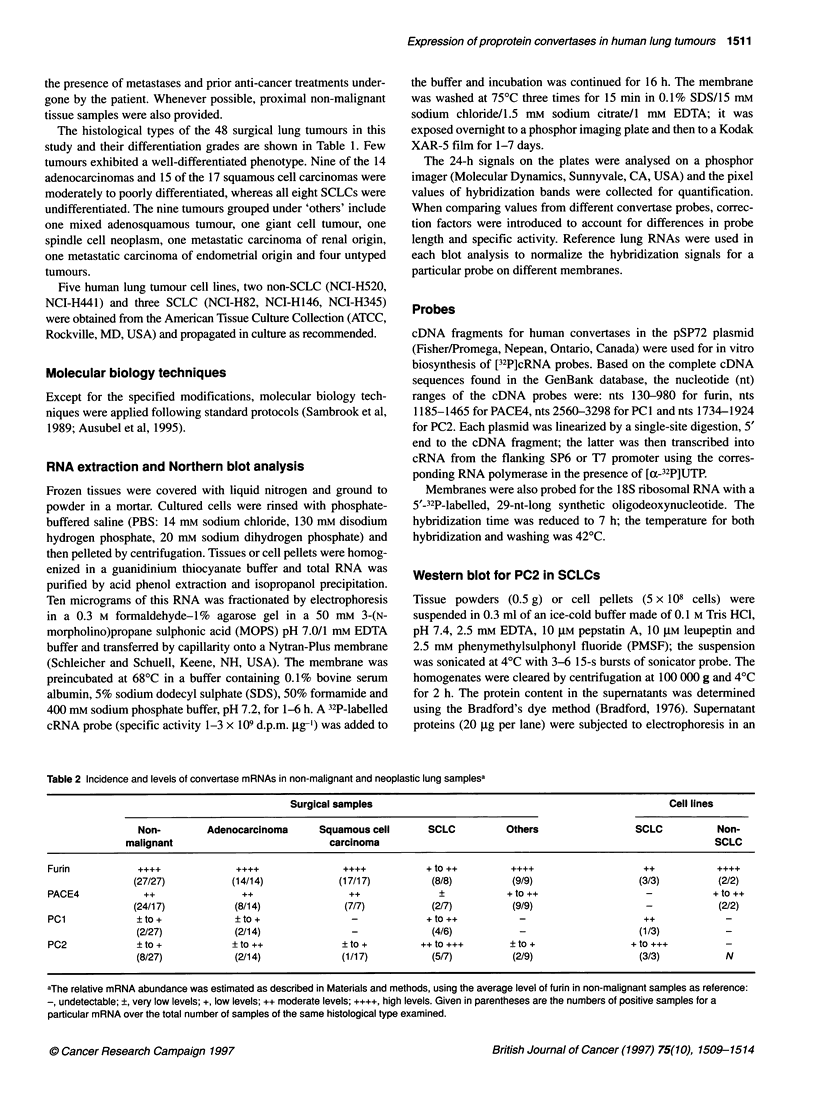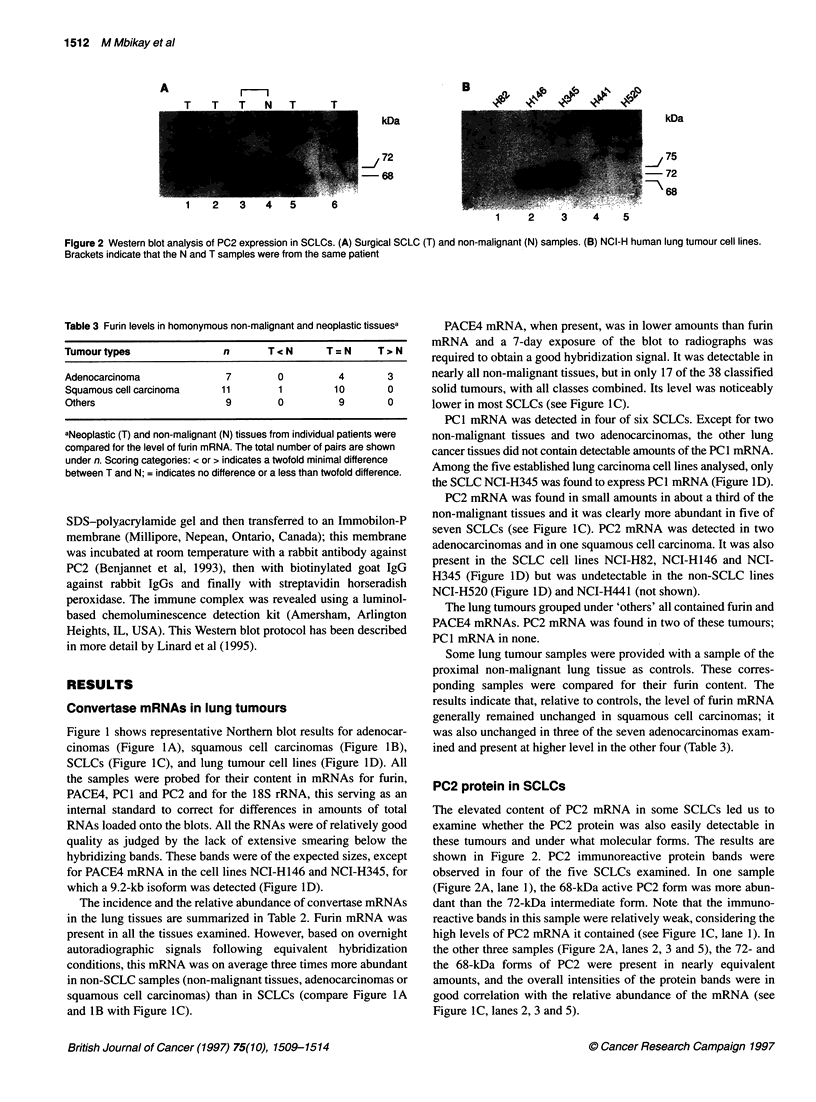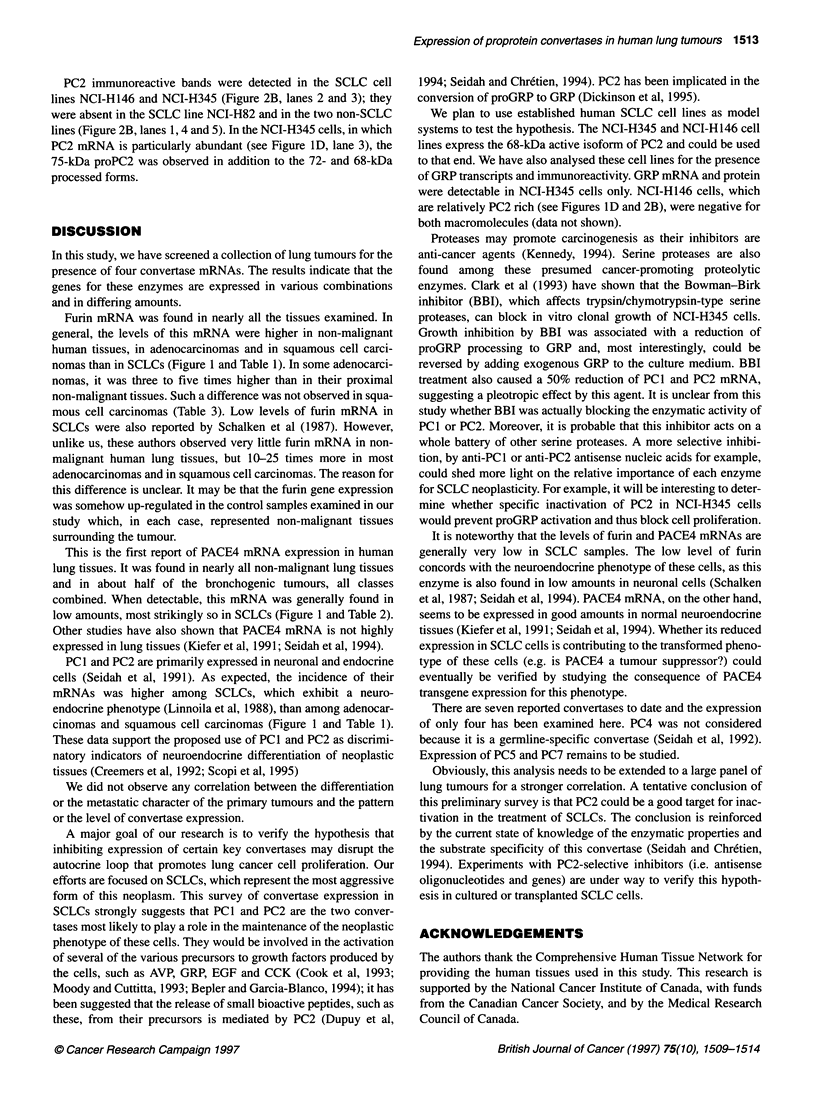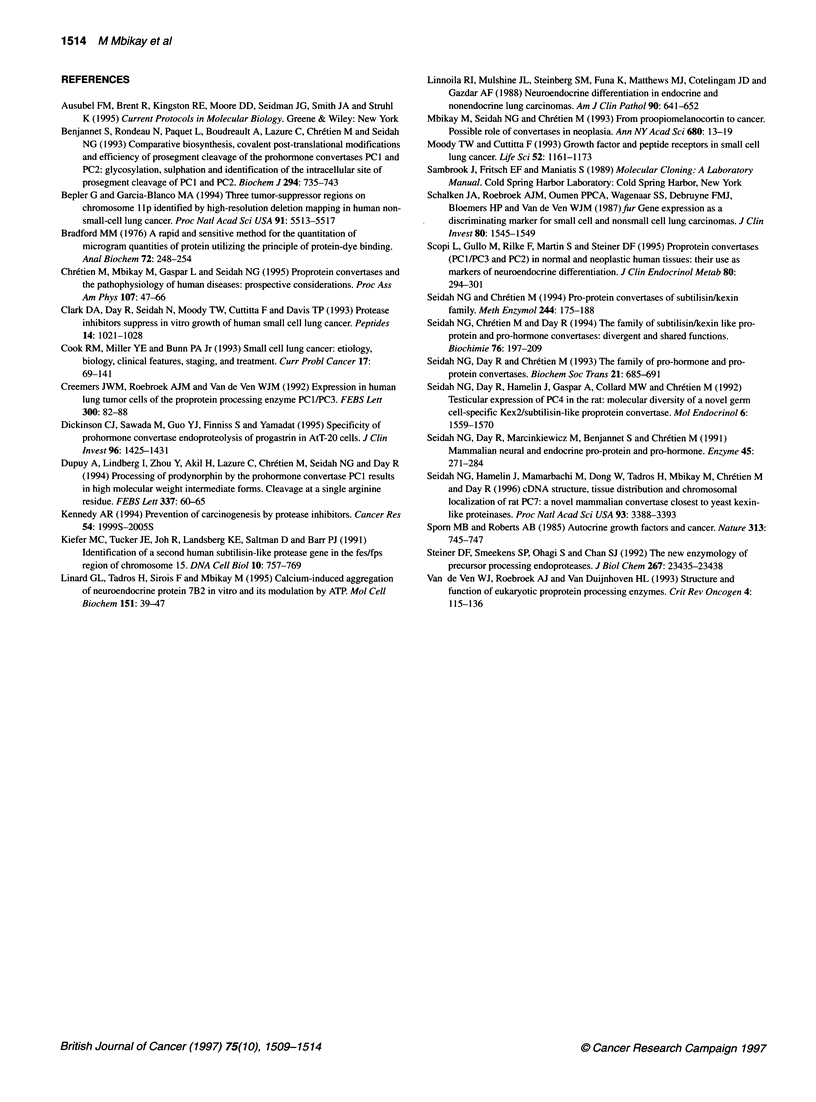# Comparative analysis of expression of the proprotein convertases furin, PACE4, PC1 and PC2 in human lung tumours.

**DOI:** 10.1038/bjc.1997.258

**Published:** 1997

**Authors:** M. Mbikay, F. Sirois, J. Yao, N. G. Seidah, M. ChrÃ©tien

**Affiliations:** Institut de Recherches Cliniques de MontrÃ©al, UniversitÃ© de MontrÃ©al, QuÃ©bec, Canada.

## Abstract

**Images:**


British Journal of Cancer (1997) 75(10), 1509–1514
© 1997 Cancer Research Campaign

### Comparative analysis of expression of the proprotein convertases furin, PACE4, PC1 and PC2 in human lung tumours

**M Mbikay, F Sirois, J Yao, NG Seidah and M Chrétien**

Institut de Recherches Cliniques de Montréal, Université de Montréal, 110 avenue des Pins Ouest Montréal, Québec, Canada H2W 1R7

**Summary** Proprotein convertases mediate the production of a variety of peptidic mitogens by limited proteolysis of their precursors. These proteases may also participate in the autocrine production of such mitogens by cancer cells and thus contribute to the unchecked proliferation of these cells. As a step towards defining this contribution, we have examined the levels of four convertase mRNAs in human lung neoplasms using semiquantitative Northern blot analysis. Furin mRNA was expressed in all the tumours; its level in squamous cell carcinomas and adenocarcinomas was on average about threefold higher than in small-cell lung carcinomas (SCLCs). PACE4 transcripts were detected in eight of 14 adenocarcinomas and in seven of 17 squamous cell carcinomas; they were detectable in only two of seven SCLCs. PC1 mRNA was undetected in squamous cell carcinomas and in all but two adenocarcinomas; it was present in four of six SCLCs. PC2 mRNA was found in two adenocarcinomas, in one squamous cell carcinoma and in five of seven SCLCs. This preliminary survey indicates that SCLCs often carry more mRNA for the endocrine convertases PC1 and PC2 and less mRNA for the more ubiquitous furin and PACE4, suggesting inverse roles of these convertases in the development of this neoplasm.

**Keywords:** convertases; proteases; lung cancer; mRNA

Neoplastic cells often express genes for peptidic growth factors and their receptors, thus creating an autocrine loop that promotes their proliferation (Sporn and Roberts, 1985). Some lung cancer cells produce one or several of the following mitogenic peptides: arginine vasopressin (AVP), neurotensin, ciliary neurotrophic factor (CNTF), gastrin-releasing peptide (GRP), cholecystokinin (CCK), neuromedin B (NMB), epidermal growth factors (EGFs) and galanin (Cook et al, 1993; Moody and Cuttitta, 1993; Bepler and Garcia-Blanco, 1994). These peptides derive from the proteolytic cleavage of their inactive precursors at sites recognized by proprotein convertases (PC).

PCs constitute a family of mammalian serine proteases, related to bacterial subtilisins and to the yeast kexin. Seven of them have been identified in recent years, namely furin, PACE4, PC1 (also called PC3), PC2, PC4, PC5 (also called PC6) and PC7. These enzymes cleave their substrates after selected basic amino acids, mostly after pairs of such residues. Besides growth factors, other potential and proven substrates include precursors to hormones, cell-surface receptors and viral glycoproteins (reviewed in Steiner et al, 1992; Seidah et al, 1993, 1994, 1996; Van de Ven et al, 1993).

Thus, PCs may be intimately involved in the production of many signalling molecules which, when abnormally expressed in lung cells, could lead to their neoplastic transformation. If, as we have hypothesized (Mbikay et al, 1993; Chrétien et al, 1995), these enzymes are inactivated somehow in these cancer cells, the cells would be unable to process a battery of endogenous mitogen precursors to active forms and lose their transformed phenotype as a consequence.

*Received 2 July 1996*
*Revised 1 November 1996*
*Accepted 18 November 1996*

*Correspondence to:* M Mbikay

Before a rational therapeutic approach based on this concept can be devised, it is important to determine which PCs are associated with the major histological types of lung cancer. In this work, we have examined a collection of lung carcinomas for the presence of detectable levels of furin, PACE4, PC1 and PC2 mRNAs.

### MATERIALS AND METHODS

### Tissues and cell lines

Surgical lung tumours were obtained from the National Cancer Institute-sponsored Cooperative Human Tissue Network (CHTN, Columbus, OH, USA). The tissues were collected at surgery and were immediately frozen on dry ice and shipped as such. They were then stored at –80°C. Most of the samples were provided with a detailed pathology report, including the histological types,

**Table 1** Differentiation grades of lung neoplastic samples[a]

| Lung tumour type | n | W | W–M | M | M–P | P | U | ? |
|---|---|---|---|---|---|---|---|---|
| Adenocarcinoma | 14 | 2 | 2 (1) | 5 (3) | 1 | 3 (2) | 0 | 1 |
| Squamous cell carcinoma | 17 | 2 (1) | 2 | 3 (2) | 3 (2) | 9 (1) | 1 (1) | 0 |
| Small-cell carcinoma | 8 | 0 | 0 | 0 | 0 | 0 | 8 | 0 |
| Others | 9 | 0 | 0 | 0 | 0 | 1 | 0 | 8 |

[a]Tumour differentiation grades were obtained from the pathology reports provided with the tissue samples. *n*, number of patients; W, well differentiated; W–M, well to moderately differentiated; M, moderately differentiated; M–P, moderately to poorly differentiated; P, poorly differentiated; U, undifferentiated;?, unknown grades (no report). The number of patients in whom lymph node metastases were observed are indicated in parentheses. Note that all non-SCLC tumours were from primary sites. All SCLCs were metastases.

**Figure 1** Representative Northern blot autoradiograms. Panels show hybridization signals obtained from various non-malignant (N) and malignant (T) lung samples probed for the convertase mRNA specified at the left. The lowest panel shows signals from the 18S rRNA probe, which was used as an internal standard to normalize the convertase mRNA signals. Brackets indicate that the N and T samples were from the same patient

*© Cancer Research Campaign 1997*

the presence of metastases and prior anti-cancer treatments undergone by the patient. Whenever possible, proximal non-malignant tissue samples were also provided.

The histological types of the 48 surgical lung tumours in this study and their differentiation grades are shown in Table 1. Few tumours exhibited a well-differentiated phenotype. Nine of the 14 adenocarcinomas and 15 of the 17 squamous cell carcinomas were moderately to poorly differentiated, whereas all eight SCLCs were undifferentiated. The nine tumours grouped under 'others' include one mixed adenosquamous tumour, one giant cell tumour, one spindle cell neoplasm, one metastatic carcinoma of renal origin, one metastatic carcinoma of endometrial origin and four untyped tumours.

Five human lung tumour cell lines, two non-SCLC (NCI-H520, NCI-H441) and three SCLC (NCI-H82, NCI-H146, NCI-H345) were obtained from the American Tissue Culture Collection (ATCC, Rockville, MD, USA) and propagated in culture as recommended.

### Molecular biology techniques

Except for the specified modifications, molecular biology techniques were applied following standard protocols (Sambrook et al, 1989; Ausubel et al, 1995).

### RNA extraction and Northern blot analysis

Frozen tissues were covered with liquid nitrogen and ground to powder in a mortar. Cultured cells were rinsed with phosphate-buffered saline (PBS: 14 mM sodium chloride, 130 mM disodium hydrogen phosphate, 20 mM sodium dihydrogen phosphate) and then pelleted by centrifugation. Tissues or cell pellets were homogenized in a guanidinium thiocyanate buffer and total RNA was purified by acid phenol extraction and isopropanol precipitation. Ten micrograms of this RNA was fractionated by electrophoresis in a 0.3 M formaldehyde–1% agarose gel in a 50 mM 3-(N-morpholino)propane sulphonic acid (MOPS) pH 7.0/1 mM EDTA buffer and transferred by capillarity onto a Nytran-Plus membrane (Schleicher and Schuell, Keene, NH, USA). The membrane was preincubated at 68°C in a buffer containing 0.1% bovine serum albumin, 5% sodium dodecyl sulphate (SDS), 50% formamide and 400 mM sodium phosphate buffer, pH 7.2, for 1–6 h. A $^{32}P$-labelled cRNA probe (specific activity $1–3 \times 10^9$ d.p.m. $\mu g^{-1}$) was added to the buffer and incubation was continued for 16 h. The membrane was washed at 75°C three times for 15 min in 0.1% SDS/15 mM sodium chloride/1.5 mM sodium citrate/1 mM EDTA; it was exposed overnight to a phosphor imaging plate and then to a Kodak XAR-5 film for 1–7 days.

The 24-h signals on the plates were analysed on a phosphor imager (Molecular Dynamics, Sunnyvale, CA, USA) and the pixel values of hybridization bands were collected for quantification. When comparing values from different convertase probes, correction factors were introduced to account for differences in probe length and specific activity. Reference lung RNAs were used in each blot analysis to normalize the hybridization signals for a particular probe on different membranes.

### Probes cDNA fragments for human convertases in the pSP72 plasmid (Fisher/Promega, Nepean, Ontario, Canada) were used for in vitro biosynthesis of [$^{32}P$]cRNA probes. Based on the complete cDNA sequences found in the GenBank database, the nucleotide (nt) ranges of the cDNA probes were: nts 130–980 for furin, nts 1185–1465 for PACE4, nts 2560–3298 for PC1 and nts 1734–1924 for PC2. Each plasmid was linearized by a single-site digestion, 5′ end to the cDNA fragment; the latter was then transcribed into cRNA from the flanking SP6 or T7 promoter using the corresponding RNA polymerase in the presence of [α-$^{32}P$]UTP.

Membranes were also probed for the 18S ribosomal RNA with a 5′-$^{32}P$-labelled, 29-nt-long synthetic oligodeoxynucleotide. The hybridization time was reduced to 7 h; the temperature for both hybridization and washing was 42°C.

### Western blot for PC2 in SCLCs

Tissue powders (0.5 g) or cell pellets ($5 \times 10^8$ cells) were suspended in 0.3 ml of an ice-cold buffer made of 0.1 M Tris HCl, pH 7.4, 2.5 mM EDTA, 10 μM pepstatin A, 10 μM leupeptin and 2.5 mM phenymethylsulphonyl fluoride (PMSF); the suspension was sonicated at 4°C with 3–6 15-s bursts of sonicator probe. The homogenates were cleared by centrifugation at 100 000 g and 4°C for 2 h. The protein content in the supernatants was determined using the Bradford's dye method (Bradford, 1976). Supernatant proteins (20 μg per lane) were subjected to electrophoresis in an

**Table 2** Incidence and levels of convertase mRNAs in non-malignant and neoplastic lung samples[a]

| | Surgical samples | | | | | Cell lines | |
|---|---|---|---|---|---|---|---|
| | Non-malignant | Adenocarcinoma | Squamous cell carcinoma | SCLC | Others | SCLC | Non-SCLC |
| Furin | ++++ | ++++ | ++++ | + to ++ | ++++ | ++ | ++++ |
| | (27/27) | (14/14) | (17/17) | (8/8) | (9/9) | (3/3) | (2/2) |
| PACE4 | ++ | ++ | ++ | ± | + to ++ | – | + to ++ |
| | (24/17) | (8/14) | (7/7) | (2/7) | (9/9) | – | (2/2) |
| PC1 | ± to + | ± to + | – | + to ++ | – | ++ | – |
| | (2/27) | (2/14) | – | (4/6) | – | (1/3) | – |
| PC2 | ± to + | ± to ++ | ± to + | ++ to +++ | ± to + | + to +++ | – |
| | (8/27) | (2/14) | (1/17) | (5/7) | (2/9) | (3/3) | *N* |

[a]The relative mRNA abundance was estimated as described in Materials and methods, using the average level of furin in non-malignant samples as reference: –, undetectable; ±, very low levels; +, low levels; ++ moderate levels; ++++, high levels. Given in parentheses are the numbers of positive samples for a particular mRNA over the total number of samples of the same histological type examined.

© Cancer Research Campaign 1997 

**Figure 2** Western blot analysis of PC2 expression in SCLCs. (**A**) Surgical SCLC (T) and non-malignant (N) samples. (**B**) NCI-H human lung tumour cell lines. Brackets indicate that the N and T samples were from the same patient

**Table 3** Furin levels in homonymous non-malignant and neoplastic tissues[a]

| Tumour types | *n* | T < N | T = N | T > N |
|---|---|---|---|---|
| Adenocarcinoma | 7 | 0 | 4 | 3 |
| Squamous cell carcinoma | 11 | 1 | 10 | 0 |
| Others | 9 | 0 | 9 | 0 |

[a]Neoplastic (T) and non-malignant (N) tissues from individual patients were compared for the level of furin mRNA. The total number of pairs are shown under *n*. Scoring categories: < or > indicates a twofold minimal difference between T and N; = indicates no difference or a less than twofold difference.

SDS–polyacrylamide gel and then transferred to an Immobilon-P membrane (Millipore, Nepean, Ontario, Canada); this membrane was incubated at room temperature with a rabbit antibody against PC2 (Benjannet et al, 1993), then with biotinylated goat IgG against rabbit IgGs and finally with streptavidin horseradish peroxidase. The immune complex was revealed using a luminol-based chemoluminescence detection kit (Amersham, Arlington Heights, IL, USA). This Western blot protocol has been described in more detail by Linard et al (1995).

### RESULTS

### Convertase mRNAs in lung tumours

Figure 1 shows representative Northern blot results for adenocarcinomas (Figure 1A), squamous cell carcinomas (Figure 1B), SCLCs (Figure 1C), and lung tumour cell lines (Figure 1D). All the samples were probed for their content in mRNAs for furin, PACE4, PC1 and PC2 and for the 18S rRNA, this serving as an internal standard to correct for differences in amounts of total RNAs loaded onto the blots. All the RNAs were of relatively good quality as judged by the lack of extensive smearing below the hybridizing bands. These bands were of the expected sizes, except for PACE4 mRNA in the cell lines NCI-H146 and NCI-H345, for which a 9.2-kb isoform was detected (Figure 1D).

The incidence and the relative abundance of convertase mRNAs in the lung tissues are summarized in Table 2. Furin mRNA was present in all the tissues examined. However, based on overnight autoradiographic signals following equivalent hybridization conditions, this mRNA was on average three times more abundant in non-SCLC samples (non-malignant tissues, adenocarcinomas or squamous cell carcinomas) than in SCLCs (compare Figure 1A and 1B with Figure 1C).

PACE4 mRNA, when present, was in lower amounts than furin mRNA and a 7-day exposure of the blot to radiographs was required to obtain a good hybridization signal. It was detectable in nearly all non-malignant tissues, but in only 17 of the 38 classified solid tumours, with all classes combined. Its level was noticeably lower in most SCLCs (see Figure 1C).

PC1 mRNA was detected in four of six SCLCs. Except for two non-malignant tissues and two adenocarcinomas, the other lung cancer tissues did not contain detectable amounts of the PC1 mRNA. Among the five established lung carcinoma cell lines analysed, only the SCLC NCI-H345 was found to express PC1 mRNA (Figure 1D).

PC2 mRNA was found in small amounts in about a third of the non-malignant tissues and it was clearly more abundant in five of seven SCLCs (see Figure 1C). PC2 mRNA was detected in two adenocarcinomas and in one squamous cell carcinoma. It was also present in the SCLC cell lines NCI-H82, NCI-H146 and NCI-H345 (Figure 1D) but was undetectable in the non-SCLC lines NCI-H520 (Figure 1D) and NCI-H441 (not shown).

The lung tumours grouped under 'others' all contained furin and PACE4 mRNAs. PC2 mRNA was found in two of these tumours; PC1 mRNA in none.

Some lung tumour samples were provided with a sample of the proximal non-malignant lung tissue as controls. These corresponding samples were compared for their furin content. The results indicate that, relative to controls, the level of furin mRNA generally remained unchanged in squamous cell carcinomas; it was also unchanged in three of the seven adenocarcinomas examined and present at higher level in the other four (Table 3).

### PC2 protein in SCLCs

The elevated content of PC2 mRNA in some SCLCs led us to examine whether the PC2 protein was also easily detectable in these tumours and under what molecular forms. The results are shown in Figure 2. PC2 immunoreactive protein bands were observed in four of the five SCLCs examined. In one sample (Figure 2A, lane 1), the 68-kDa active PC2 form was more abundant than the 72-kDa intermediate form. Note that the immunoreactive bands in this sample were relatively weak, considering the high levels of PC2 mRNA it contained (see Figure 1C, lane 1). In the other three samples (Figure 2A, lanes 2, 3 and 5), the 72- and the 68-kDa forms of PC2 were present in nearly equivalent amounts, and the overall intensities of the protein bands were in good correlation with the relative abundance of the mRNA (see Figure 1C, lanes 2, 3 and 5).

 *© Cancer Research Campaign 1997*

PC2 immunoreactive bands were detected in the SCLC cell lines NCI-H146 and NCI-H345 (Figure 2B, lanes 2 and 3); they were absent in the SCLC line NCI-H82 and in the two non-SCLC lines (Figure 2B, lanes 1, 4 and 5). In the NCI-H345 cells, in which PC2 mRNA is particularly abundant (see Figure 1D, lane 3), the 75-kDa proPC2 was observed in addition to the 72- and 68-kDa processed forms.

### DISCUSSION

In this study, we have screened a collection of lung tumours for the presence of four convertase mRNAs. The results indicate that the genes for these enzymes are expressed in various combinations and in differing amounts.

Furin mRNA was found in nearly all the tissues examined. In general, the levels of this mRNA were higher in non-malignant human tissues, in adenocarcinomas and in squamous cell carcinomas than in SCLCs (Figure 1 and Table 1). In some adenocarcinomas, it was three to five times higher than in their proximal non-malignant tissues. Such a difference was not observed in squamous cell carcinomas (Table 3). Low levels of furin mRNA in SCLCs were also reported by Schalken et al (1987). However, unlike us, these authors observed very little furin mRNA in non-malignant human lung tissues, but 10–25 times more in most adenocarcinomas and in squamous cell carcinomas. The reason for this difference is unclear. It may be that the furin gene expression was somehow up-regulated in the control samples examined in our study which, in each case, represented non-malignant tissues surrounding the tumour.

This is the first report of PACE4 mRNA expression in human lung tissues. It was found in nearly all non-malignant lung tissues and in about half of the bronchogenic tumours, all classes combined. When detectable, this mRNA was generally found in low amounts, most strikingly so in SCLCs (Figure 1 and Table 2). Other studies have also shown that PACE4 mRNA is not highly expressed in lung tissues (Kiefer et al, 1991; Seidah et al, 1994).

PC1 and PC2 are primarily expressed in neuronal and endocrine cells (Seidah et al, 1991). As expected, the incidence of their mRNAs was higher among SCLCs, which exhibit a neuroendocrine phenotype (Linnoila et al, 1988), than among adenocarcinomas and squamous cell carcinomas (Figure 1 and Table 1). These data support the proposed use of PC1 and PC2 as discriminatory indicators of neuroendocrine differentiation of neoplastic tissues (Creemers et al, 1992; Scopi et al, 1995)

We did not observe any correlation between the differentiation or the metastatic character of the primary tumours and the pattern or the level of convertase expression.

A major goal of our research is to verify the hypothesis that inhibiting expression of certain key convertases may disrupt the autocrine loop that promotes lung cancer cell proliferation. Our efforts are focused on SCLCs, which represent the most aggressive form of this neoplasm. This survey of convertase expression in SCLCs strongly suggests that PC1 and PC2 are the two convertases most likely to play a role in the maintenance of the neoplastic phenotype of these cells. They would be involved in the activation of several of the various precursors to growth factors produced by the cells, such as AVP, GRP, EGF and CCK (Cook et al, 1993; Moody and Cuttitta, 1993; Bepler and Garcia-Blanco, 1994); it has been suggested that the release of small bioactive peptides, such as these, from their precursors is mediated by PC2 (Dupuy et al, 1994; Seidah and Chrétien, 1994). PC2 has been implicated in the conversion of proGRP to GRP (Dickinson et al, 1995).

We plan to use established human SCLC cell lines as model systems to test the hypothesis. The NCI-H345 and NCI-H146 cell lines express the 68-kDa active isoform of PC2 and could be used to that end. We have also analysed these cell lines for the presence of GRP transcripts and immunoreactivity. GRP mRNA and protein were detectable in NCI-H345 cells only. NCI-H146 cells, which are relatively PC2 rich (see Figures 1D and 2B), were negative for both macromolecules (data not shown).

Proteases may promote carcinogenesis as their inhibitors are anti-cancer agents (Kennedy, 1994). Serine proteases are also found among these presumed cancer-promoting proteolytic enzymes. Clark et al (1993) have shown that the Bowman–Birk inhibitor (BBI), which affects trypsin/chymotrypsin-type serine proteases, can block in vitro clonal growth of NCI-H345 cells. Growth inhibition by BBI was associated with a reduction of proGRP processing to GRP and, most interestingly, could be reversed by adding exogenous GRP to the culture medium. BBI treatment also caused a 50% reduction of PC1 and PC2 mRNA, suggesting a pleotropic effect by this agent. It is unclear from this study whether BBI was actually blocking the enzymatic activity of PC1 or PC2. Moreover, it is probable that this inhibitor acts on a whole battery of other serine proteases. A more selective inhibition, by anti-PC1 or anti-PC2 antisense nucleic acids for example, could shed more light on the relative importance of each enzyme for SCLC neoplasticity. For example, it will be interesting to determine whether specific inactivation of PC2 in NCI-H345 cells would prevent proGRP activation and thus block cell proliferation.

It is noteworthy that the levels of furin and PACE4 mRNAs are generally very low in SCLC samples. The low level of furin concords with the neuroendocrine phenotype of these cells, as this enzyme is also found in low amounts in neuronal cells (Schalken et al, 1987; Seidah et al, 1994). PACE4 mRNA, on the other hand, seems to be expressed in good amounts in normal neuroendocrine tissues (Kiefer et al, 1991; Seidah et al, 1994). Whether its reduced expression in SCLC cells is contributing to the transformed phenotype of these cells (e.g. is PACE4 a tumour suppressor?) could eventually be verified by studying the consequence of PACE4 transgene expression for this phenotype.

There are seven reported convertases to date and the expression of only four has been examined here. PC4 was not considered because it is a germline-specific convertase (Seidah et al, 1992). Expression of PC5 and PC7 remains to be studied.

Obviously, this analysis needs to be extended to a large panel of lung tumours for a stronger correlation. A tentative conclusion of this preliminary survey is that PC2 could be a good target for inactivation in the treatment of SCLCs. The conclusion is reinforced by the current state of knowledge of the enzymatic properties and the substrate specificity of this convertase (Seidah and Chrétien, 1994). Experiments with PC2-selective inhibitors (i.e. antisense oligonucleotides and genes) are under way to verify this hypothesis in cultured or transplanted SCLC cells.

### ACKNOWLEDGEMENTS

The authors thank the Comprehensive Human Tissue Network for providing the human tissues used in this study. This research is supported by the National Cancer Institute of Canada, with funds from the Canadian Cancer Society, and by the Medical Research Council of Canada.

© Cancer Research Campaign 1997

### REFERENCES

Ausubel FM, Brent R, Kingston RE, Moore DD, Seidman JG, Smith JA and Struhl K (1995) *Current Protocols in Molecular Biology*. Greene & Wiley: New York

Benjannet S, Rondeau N, Paquet L, Boudreault A, Lazure C, Chrétien M and Seidah NG (1993) Comparative biosynthesis, covalent post-translational modifications and efficiency of prosegment cleavage of the prohormone convertases PC1 and PC2: glycosylation, sulphation and identification of the intracellular site of prosegment cleavage of PC1 and PC2. *Biochem J* **294**: 735–743

Bepler G and Garcia-Blanco MA (1994) Three tumor-suppressor regions on chromosome 11p identified by high-resolution deletion mapping in human non-small-cell lung cancer. *Proc Natl Acad Sci USA* **91**: 5513–5517

Bradford MM (1976) A rapid and sensitive method for the quantitation of microgram quantities of protein utilizing the principle of protein-dye binding. *Anal Biochem* **72**: 248–254

Chrétien M, Mbikay M, Gaspar L and Seidah NG (1995) Proprotein convertases and the pathophysiology of human diseases: prospective considerations. *Proc Ass Am Phys* **107**: 47–66

Clark DA, Day R, Seidah N, Moody TW, Cuttitta F and Davis TP (1993) Protease inhibitors suppress in vitro growth of human small cell lung cancer. *Peptides* **14**: 1021–1028

Cook RM, Miller YE and Bunn PA Jr (1993) Small cell lung cancer: etiology, biology, clinical features, staging, and treatment. *Curr Probl Cancer* **17**: 69–141

Creemers JWM, Roebroek AJM and Van de Ven WJM (1992) Expression in human lung tumor cells of the proprotein processing enzyme PC1/PC3. *FEBS Lett* **300**: 82–88

Dickinson CJ, Sawada M, Guo YJ, Finniss S and Yamadat (1995) Specificity of prohormone convertase endoproteolysis of progastrin in AtT-20 cells. *J Clin Invest* **96**: 1425–1431

Dupuy A, Lindberg I, Zhou Y, Akil H, Lazure C, Chrétien M, Seidah NG and Day R (1994) Processing of prodynorphin by the prohormone convertase PC1 results in high molecular weight intermediate forms. Cleavage at a single arginine residue. *FEBS Lett* **337**: 60–65

Kennedy AR (1994) Prevention of carcinogenesis by protease inhibitors. *Cancer Res* **54**: 1999S–2005S

Kiefer MC, Tucker JE, Joh R, Landsberg KE, Saltman D and Barr PJ (1991) Identification of a second human subtilisin-like protease gene in the fes/fps region of chromosome 15. *DNA Cell Biol* **10**: 757–769

Linard GL, Tadros H, Sirois F and Mbikay M (1995) Calcium-induced aggregation of neuroendocrine protein 7B2 in vitro and its modulation by ATP. *Mol Cell Biochem* **151**: 39–47

Linnoila RI, Mulshine JL, Steinberg SM, Funa K, Matthews MJ, Cotelingam JD and Gazdar AF (1988) Neuroendocrine differentiation in endocrine and nonendocrine lung carcinomas. *Am J Clin Pathol* **90**: 641–652

Mbikay M, Seidah NG and Chrétien M (1993) From proopiomelanocortin to cancer. Possible role of convertases in neoplasia. *Ann NY Acad Sci* **680**: 13–19

Moody TW and Cuttitta F (1993) Growth factor and peptide receptors in small cell lung cancer. *Life Sci* **52**: 1161–1173

Sambrook J, Fritsch EF and Maniatis S (1989) *Molecular Cloning: A Laboratory Manual*. Cold Spring Harbor Laboratory: Cold Spring Harbor, New York

Schalken JA, Roebroek AJM, Oumen PPCA, Wagenaar SS, Debruyne FMJ, Bloemers HP and Van de Ven WJM (1987) *fur* Gene expression as a discriminating marker for small cell and nonsmall cell lung carcinomas. *J Clin Invest* **80**: 1545–1549

Scopi L, Gullo M, Rilke F, Martin S and Steiner DF (1995) Proprotein convertases (PC1/PC3 and PC2) in normal and neoplastic human tissues: their use as markers of neuroendocrine differentiation. *J Clin Endocrinol Metab* **80**: 294–301

Seidah NG and Chrétien M (1994) Pro-protein convertases of subtilisin/kexin family. *Meth Enzymol* **244**: 175–188

Seidah NG, Chrétien M and Day R (1994) The family of subtilisin/kexin like pro-protein and pro-hormone convertases: divergent and shared functions. *Biochimie* **76**: 197–209

Seidah NG, Day R and Chrétien M (1993) The family of pro-hormone and pro-protein convertases. *Biochem Soc Trans* **21**: 685–691

Seidah NG, Day R, Hamelin J, Gaspar A, Collard MW and Chrétien M (1992) Testicular expression of PC4 in the rat: molecular diversity of a novel germ cell-specific Kex2/subtilisin-like proprotein convertase. *Mol Endocrinol* **6**: 1559–1570

Seidah NG, Day R, Marcinkiewicz M, Benjannet S and Chrétien M (1991) Mammalian neural and endocrine pro-protein and pro-hormone. *Enzyme* **45**: 271–284

Seidah NG, Hamelin J, Mamarbachi M, Dong W, Tadros H, Mbikay M, Chrétien M and Day R (1996) cDNA structure, tissue distribution and chromosomal localization of rat PC7: a novel mammalian convertase closest to yeast kexin-like proteinases. *Proc Natl Acad Sci USA* **93**: 3388–3393

Sporn MB and Roberts AB (1985) Autocrine growth factors and cancer. *Nature* **313**: 745–747

Steiner DF, Smeekens SP, Ohagi S and Chan SJ (1992) The new enzymology of precursor processing endoproteases. *J Biol Chem* **267**: 23435–23438

Van de Ven WJ, Roebroek AJ and Van Duijnhoven HL (1993) Structure and function of eukaryotic proprotein processing enzymes. *Crit Rev Oncogen* **4**: 115–136

© Cancer Research Campaign 1997